# Whole-Genome Analysis of G2P[4] Rotavirus Strains in China in 2022 and Comparison of Their Antigenic Epitopes with Vaccine Strains

**DOI:** 10.3390/v17030326

**Published:** 2025-02-26

**Authors:** Ruyi Che, Jiaxin Fan, Guangping Xiong, Lingshan Kong, Mengjie Dong, Yi Li, Peng Wang, Jianguang Fu, Zhenlu Sun, Song Liu, Caixia Li, Xuan Feng, Xiaoman Sun, Dandi Li, Zhaojun Duan

**Affiliations:** 1 National Key Laboratory of Intelligent Tracking and Forecasting for Infectious Diseases (NITFID), NHC Key Laboratory for Medical Virology and Viral Disease, National Institute for Viral Disease Control and Prevention, Chinese Center for Disease Control and Prevention, Beijing 102200, China; cheruyi2022@163.com (R.C.);; 2 Henan Provincial Center for Disease Control and Prevention, Zhengzhou 450016, China; 3 Gansu Provincial Center for Disease Control and Prevention, Lanzhou 730000, China; 4 Jiangsu Provincial Center for Disease Control and Prevention, Nanjing 210009, China; 5 Yantai Center for Disease Control and Prevention, Yantai 264003, China; 6 Shandong Center for Disease Control and Prevention, Jinan 266000, China; 7 Guangdong Provincial Center for Disease Control and Prevention, Guangzhou 511430, China; 8 Shaanxi Provincial Center for Disease Control and Prevention, Xi’an 710054, China

**Keywords:** rotavirus, G2P[4], whole-genome analysis, antigenic epitope, China

## Abstract

Group A rotavirus (RVA) is the leading cause of acute gastroenteritis in infants and young children worldwide. To elucidate the molecular epidemiology of G2P[4] rotavirus in China and the protective effects of vaccines, whole-genome analysis of 13 G2P[4] RVA strains collected from China in 2022 was performed. Twelve strains possessed the typical DS-1-like genome constellation G2-P[4]-I2-R2-C2-M2-A2-N2-T2-E2-H2. Only GS2265 possessed the genome constellation G2-P[4]-12-R2-C2-M2-A2-N2-T2-E1-H2. With the exception of the NSP4 segment of GS2265, all other sequences of the 13 G2P[4] RVA strains clustered within the same lineage on phylogenetic analysis. However, QD2210 and SX2205 were grouped into different branches compared to the other strains. In the VP7 antigenic epitopes, four residues differed from the RotaTeq G2 strain; specifically, A87T and D96N in the 7-1a region and S213D and S242N in the 7-1b region. Comparison of the current G2P[4] RVA strains circulating in China with those circulating globally revealed a high degree of sequence identity. High genetic variability among the newly characterized G2P[4] RVA strains suggest the strains evolve fast. Finally, our data suggest that the multivalent RotaTeq vaccine could have provided better protection than the monovalent Rotarix and LLR.

## 1. Introduction

Group A rotavirus (RVA) is the leading cause of acute gastroenteritis in infants and young children worldwide. In 2016, it was estimated that 128,500 children under the age of five died from rotavirus gastroenteritis (RVGE) globally. Each year, RVGE causes approximately 453,000 deaths, with the vast majority occurring in developing countries in Asia and Sub-Saharan Africa [1]. RV is classified into nine species (A, B, C, D, F, G, H, I, and J) in the genus Rotavirus, family Sedoreoviridae [2]. RVA is a non-enveloped, double-stranded RNA virus. The virion is a three-layered particle consisting of a core, inner capsid, and outer capsid, containing the viral genome composed of 11 double-stranded RNA segments. These segments encode six structural proteins and six non-structural proteins [3]. A binary classification scheme has traditionally been used to classify RVA into G and P types based on the properties of the outer capsid proteins, VP7 and VP4 [4]. The full genome, consisting of 11 segments (VP7-VP4-VP6-VP1-VP2-VP3-NSP1-NSP2-NSP3-NSP4-NSP5/6), can be classified into genotypes as Gx-P[x]-Ix-Rx-Cx-Mx-Ax-Nx-Tx-Ex-Hx. Based on genotype, they can be classified into a Wa-like genome (G1/3/4/9/12-P[8]-I1-R1-C1-M1-A1-N1-T1-E1-H1), a DS-1-like genome (G2-P[4]-I2-R2-C2-M2-A2-N2-T2-E2-H2), and an AU-1-like genome (G3-P[9]-I3-R3-C3-M3-A3-N3-T3-E3-H3) [5,6]. In the Western Pacific Region, G9P[8] represented 40% of all genotypes, followed by G1P[8] (24%) and G2P[4] (12%) [7]. The proportion of the G2P[4] type is relatively low. However, there was an unusual increase in diarrhea caused by rotavirus type G2P[4] in Gansu Province, China, in 2022. Therefore, a genome-wide analysis of rotavirus type G2P[4] strains across the country is necessary.

Results from the Global Rotavirus Surveillance Network (GRSN) indicate that the incidence of RVGE is approximately 38% among children with acute gastroenteritis in countries without a national RVA immunization program. By contrast, in countries where the RVA vaccine has been introduced, the incidence of RVGE is about 23% [8]. Two live attenuated RVA vaccines have been approved for use in 126 countries worldwide: RotaTeq (RV5; Merck, Whitehouse Station, NJ, USA), a pentavalent (G1, G2, G3, G4, P[8]) human–bovine reassortant RVA vaccine [9]; and Rotarix (RV1; GlaxoSmithKline Biologicals, Rixensart, Belgium), a monovalent (G1P[8]) RVA vaccine derived from an attenuated human strain [10]. The Lanzhou Lamb RVA vaccine (LLR; Lanzhou Institute of Biological Products, Lanzhou, China) is a monovalent G10P[15] live attenuated vaccine used only in China [11]. The trivalent (G2, G3, G4) oral human–lamb reassortant RVA live vaccine (LLR3; Lanzhou Institute of Biological Products) was approved for use in China in April 2023 [12]. LLR3 uses the LLR vaccine strain as the parent strain, with reassortment of the VP7 gene from human RVA epidemic strains. Currently, RVA vaccines are not included in the National Immunisation Program in China, and the vaccination rate and public awareness of RVA vaccines must be improved.

The aims of present study were to conduct a full-genotype characterization of thirteen G2P[4] RVA strains in China in 2022 and to perform phylogenetic analysis to understand their genetic diversity and evolution. Comparison of the VP7 and VP4 proteins of the RVA strains with those of the vaccine strains identified potentially important antigenic differences that could facilitate the development and introduction of an RVA vaccine in China.

## 2. Materials and Methods

### 2.1. Sample Source

A total of 13 G2P[4] RVA strains were collected by the Chinese National Viral Diarrhoea Surveillance Network in 2022. (Table 1). The Chinese Centre for Disease Control and Prevention (China CDC) established national surveillance sites in 31 provinces across the country, with 1~3 sentinel hospitals selected in each province to collect clinical information and stool specimens from all hospitalized diarrheal children under 5 years of age (≤59 months of age) in that hospital. All 13 samples were obtained from children under 5 years old who were hospitalized due to acute gastroenteritis, with informed consent provided by their guardians. The included patients were all ≤25 months of age, with an average admission body temperature of 37.18 °C. On average, the patients experienced five episodes of diarrhea and two episodes of vomiting per day. In 2022, samples were collected from six provinces in China, transported via the cold chain to the National Institute for Viral Disease Control and Prevention at the China CDC, and stored at –80 °C.

### 2.2. Virus Nucleic Acid Extraction, RT-PCR and Nucleotide Sequencing

A 10% fecal suspension was prepared from each sample in phosphate-buffered saline (PBS, 0.01 mol/L, pH 7.2–7.4). After 8000× *g* centrifugation for 5 min, 100 μL of the supernatant of the stool suspension was used to determine RVA-positive samples by ELISA (Thermo Scientific™ ProSpecT™ Rotavirus Microplate Assay; Thermo Fisher Scientific, Waltham, MA, USA) in accordance with the operation manual. RNA was extracted from the samples using the Tianlong automated nucleic acid extraction system (GeneRotex 96; Xi’an Tianlong Science and Technology Co., Ltd., Xi’an, China) and stored at −80 °C until use. RNA was reverse transcribed into cDNA using a SuperScript™ III Reverse Transcriptase Kit (18080093; Invitrogen, Carlsbad, CA, USA), with conserved primers [13] targeting both ends of the RVA genome. RT-PCRs for the 11 gene segments were performed using primers described by Varghese et al. [14] (VP1, VP2, and VP3), Wang et al. [15] (NSP1, NSP2, NSP4, NSP5/6, and VP6), Magagula et al. [16] (NSP2, NSP3, NSP4, NSP5/6, and VP6), Gómara et al. [17] (VP7), and Simmonds et al. [18] (VP4). The 11 genomic segments were amplified separately using I-5™ 2x T8 High-Fidelity Master Mix (TSE111; Beijing Tsingke Biotech Co., Ltd., Beijing, China). Taq polymerase was activated for 3 min at 94 °C, followed by 35 cycles of amplification (30 s at 94 °C, 30 s at 55 °C, and 60 s at 72 °C), with a final extension for 10 min at 72 °C. The PCR products were sequenced using the Sanger method at Beijing Tsingke Biotech Co., Ltd. (Beijing, China) Nucleotide sequences were determined using an ABIPRISM 3730 automated DNA sequencer (Thermo Fisher Scientific, Waltham, MA, USA). Sequencing used the same primers as PCR reaction.

### 2.3. Sequence Analysis

Whole-genome sequences (excluding primer sequences) were assembled using SeqMan software (DNAStar 5.1). After sequencing and assembly, nearly full-length sequences (except for the 5′ and 3′ terminal sequences) were obtained. The sequencing results were genotyped using the online Basic Local Alignment Search Tool (BLAST). The sequences of 13 G2P[4] RVA strains were aligned using Clustal W. The phylogenetic trees were created using the MEGA v11.0 software based on the maximum likelihood method and selected the best-fit evolutionary model based on the corrected Bayesian information criterion value [19]. The models used in this study were T92 + G + I for VP2, VP3, VP6, and VP7; T92 + G for VP4, NSP2, and NSP5/6; T92 + I for NSP1, NSP3, and NSP4; and GTR + G + I for VP1. Branch support was estimated with 1000 bootstrap replicates, with values >70% considered significant. The lineage classification system referred to Agbemabiese et al. [20,21], Doan et al. [22], and Medeiros et al. [23]. Analysis of sequence identity was performed using MegAlign software (DNAstar 5.1).

### 2.4. Protein Model Construction and Analysis

The VP7 (Protein Data Bank [PDB]: 3FMG) and VP4 (PDB: 1KOR) structures of the G2P[4] strain were constructed using SWISS-MODEL (https://swissmodel.expasy.org/, accessed on 4 May 2024). Structural analysis was performed using PyMOL (http://www.pymol.org/pymol, accessed on 28 May 2024).

## 3. Results

### 3.1. Analysis of Whole-Genome Constellation of G2P[4] RVA Strains

Comparison of the full genomic sequences of the 11 gene segments from 13 G2P[4] RVA strains collected in China in 2022 revealed that the genotype of the GS2265 strain was G2P[4]-I2-R2-C2-M2-A2-N2-T2-E1-H2. The remaining strains exhibited the typical DS-1-like genetic backbone, with the genotype G2P[4]-I2-R2-C2-M2-A2-N2-T2-E2-H2. When compared with the other reference strains deposited in the GenBank sequence databases, the complete genotype configuration of these, except GS2265 strains, was genotypically identical in whole-genome segments to the RVA/Human-wt/JPN/Tokyo18-41/2018/G2P[4] isolated in Tokyo, Japan [24], and RVA/Human-wt/CHN/E6896/2021/G2P[4] detected in Wuhan, China (GenBank: OP850417). However, in the NSP4 segment, GS2265 is consistent with typical Wa-like strain RVA/Human-wt/CHN/20200077/2020/G3P[8], isolated in Ningxia, China (GenBank: MN106174). Comparison of the complete genome constellations of the 13 G2P[4] strains with those of the other reference strains is shown in Table 2.

### 3.2. Phylogenetic Analysis of VP7/VP4 Genes

**Figure 1:**
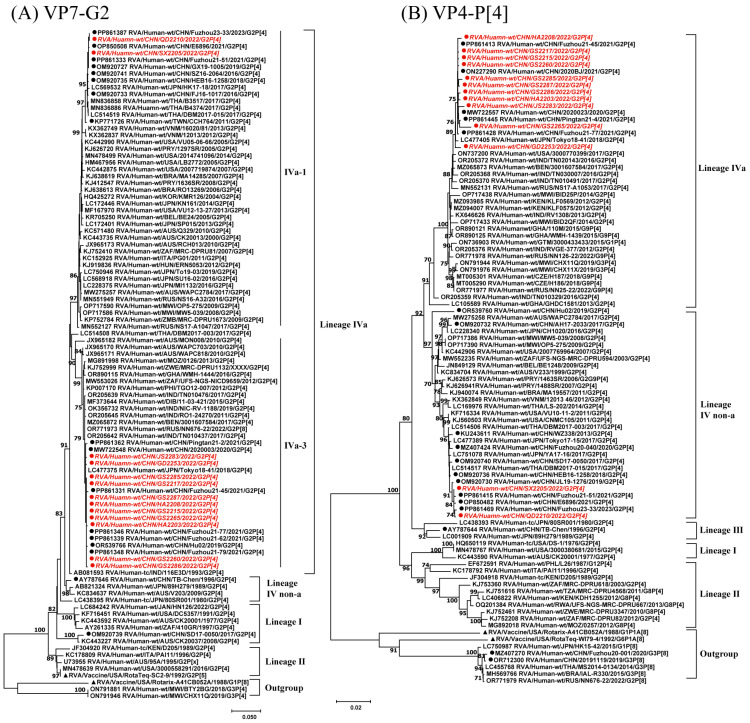
Phylogenetic analysis of the VP7 and VP4 genes of 13 G2P[4] RVA strains from China in 2022. Maximum Likelihood (ML) phylogenetic trees were constructed using MEGA v11.0, with a branch node statistical significance > 70%. ●, G2P[4] strains from this study, with sequence names in italics; ●, RVA strains from China; ▲, reference vaccine strains.

Among the 13 G2P[4] RVA strains analyzed in this study, the VP7 gene demonstrated a nucleotide sequence identity > 94.8% and an amino acid sequence identity > 94.7%. Phylogenetic analysis of the VP7 gene revealed that the G2 strains formed four lineages (I, II, IVa, and IVnon-a) (Figure 1A). All 13 strains in this study belonged to different sublineages within the same lineage. QD2210 and SX2205 were located in sublineage IVa-1 and showed high similarity (99.9%). The remaining 11 strains were grouped into sublineage IVa-3, with nucleotide identities of 99.6–100%. The RotaTeq G2 strain was situated in lineage II. QD2210 and SX2205 were closely related to G2 strains detected in Fuzhou (Fuzhou23–33, Fuzhou21–51) and Wuhan (E6896), China, between 2021 and 2023, showing an identity of 99.7–100%. The other 11 strains were grouped into sublineage IVa-3, with strains from mainland China, India, Russia, Benin, and Bangladesh. They were closely related to strains from Fujian, China (Fuzhou21–79, 21–7, 21–45, 21–62) and Japan (Tokyo18–41), with a nucleotide identity of 99.6–100%. These 11 strains were more distantly related to the earlier Chinese G2P[4] strain, TB-Chen, with an identity of 96.6–96.7% (Figure 1A).

In this study, the 13 G2P[4] RVA strains showed a nucleotide sequence identity of 96.1–99.9% and amino acid sequence identity of 95.3–99.9% in the VP4 segment. The clustering pattern of the VP4 phylogenetic tree was similar to that of VP7. The P[4] strains in the VP4 segment formed five lineages (I–IVa, IVnon-a). QD2210 and SX2205 were located in sublineage IVnon-a, showing nucleotide sequence identity of 99.9%. The remaining 11 strains clustered within sublineage IVa, with a nucleotide sequence identity of 98.9–99.9%. QD2210 and SX2205 were closely related to P[4] strains detected in Fuzhou (Fuzhou23–33), Wuhan (E6896), Jilin (JL19–1276) and Hebei (HEB16–1258), China, between 2018 and 2023, with a nucleotide sequence identity of 99.7–99.9%. The other 11 strains clustered with RVA strains detected between 2018 and 2021 in Fujian (Fuzhou21–45, Pingtan21–4), Beijing (2020BJ), Yunnan (2020023), and Japan (Tokyo18–41), with a nucleotide sequence identity > 98.9%. The sequences from this study showed greater genetic distance from early DS-1-like G2P[4] RVA strains from the USA, with an identity of 93.1–94% (Figure 1B).

### 3.3. Phylogenetic Analysis of VP1-VP3, VP6, and NSP1-NSP5 Genes

**Figure 2:**
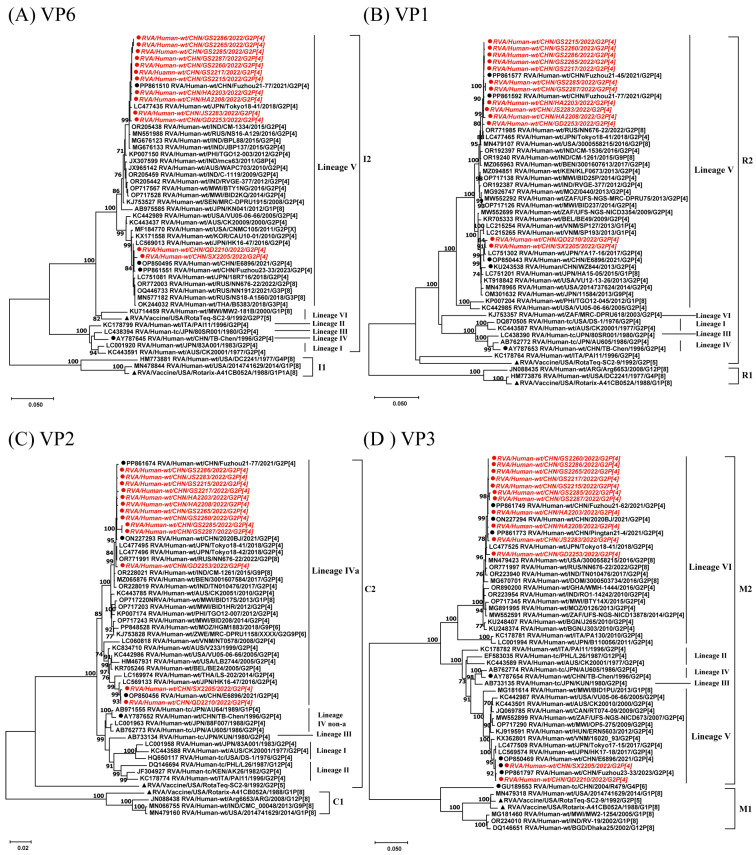
Phylogenetic analysis of the VP1–VP3 and VP6 genes of 13 G2P[4] RVA strains from China in 2022. Maximum Likelihood (ML) phylogenetic trees were constructed with branch node statistical significance > 70%. ●, G2P[4] strains from this study, with sequence names in italics; ●, RVA strains from China; ▲, reference vaccine strains. The GenBank accession numbers for each strain are listed before the sequence names.

In this study, the VP1, VP2, and VP6 genes of 13 G2P[4] RVA strains showed a nucleotide sequence identity of 96.1–100%, while VP3 showed nucleotide sequence identities ranging from 87% to 100%. Phylogenetic analysis revealed that QD2210 and SX2205 were closely related and located on a different branch from the remaining 11 RVA strains. The strain E6896, detected in Wuhan in 2021, demonstrated close genetic relationships with strains QD2210 and SX2205, with a nucleotide sequence identity of 99.6–100% across four genome segments. The other 11 strains clustered closely with strains from mainland China and Japan, such as Fuzhou21–77 and Tokyo18–41, and showed a 99.3–100% nucleotide sequence identity. For the VP3 gene, QD2210 and SX2205 are located in lineage V, while the remaining strains belong to lineage VI. Furthermore, the RotaTeq vaccine strain was located in the M1 lineage along with Rotarix. In contrast, for the VP1, VP2, and VP6 genes, all 13 G2P[4] RVA strains are of the same lineage. The RotaTeq vaccine strain belongs to the DS-1-like but is in different lineages compared to the strains in this study (Figure 2).

**Figure 3:**
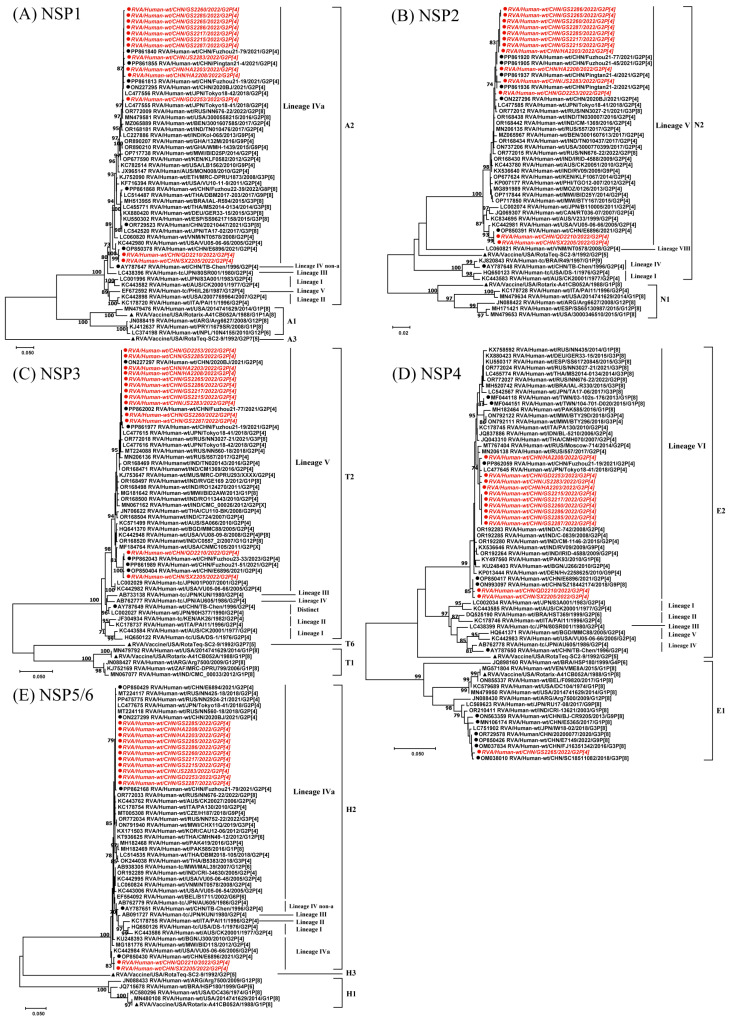
Phylogenetic analysis of the NSP1–NSP5/6 genes of 13 G2P[4] RVA strains from China in 2022. Maximum Likelihood (ML) phylogenetic trees were constructed with branch node statistical significance > 70%. ●, G2P[4] strains from this study, with sequence names in italics; ●, RVA strains from China; ▲, reference vaccine strains. The GenBank accession numbers for each strain are listed before the sequence names.

The NSP1–NSP3 and NSP5 genes exhibited nucleotide sequence identities ranging from 94.7% to 100%, while the NSP4 segment showed an identity of 82.1–100%. The phylogenetic trees of NSP1–NSP5 showed similar clustering patterns to those of VP1–VP3 and VP6. Notably, GS2265 was uniquely positioned in the E1 lineage for the NSP4 gene. This strain clustered with Wa-like strains from multiple regions in China, including Beijing, Fujian, and Sichuan, as well as strains from neighboring countries (e.g., Japan and India) during the period 2013–2022. GS2265 showed nucleotide identities of 98.8–99.9% with Chinese Wa-like strains and 98.5–99.6% nucleotide identities with Wa-like strains from neighboring countries. In the NSP4 gene, the nucleotide identity between GS2265 and other strains in this study ranged from 82.3% to 99.8%. The Wuhan E6896 strain shared 99.4–100% nucleotide identities across five genome segments with QD2210 and SX2205. Most of the other RVA strains were closely related to G2P[4] strains from mainland China and Japan. Additionally, in the NSP4 and NSP5 genes, these strains clustered with contemporary Russian strains 557 and NN2924–21, with an identity of 99.5–99.8% and 99.9–100%, respectively. The RotaTeq vaccine strains were positioned in lineages A3, T6, and H3 for the NSP1, NSP3, and NSP5 segments, respectively. In contrast, all segments of the Rotarix strain were located in lineage I (Figure 3).

### 3.4. Comparison of VP7 and VP4 Neutralizing Epitopes with Vaccine Strains

**Figure 4:**
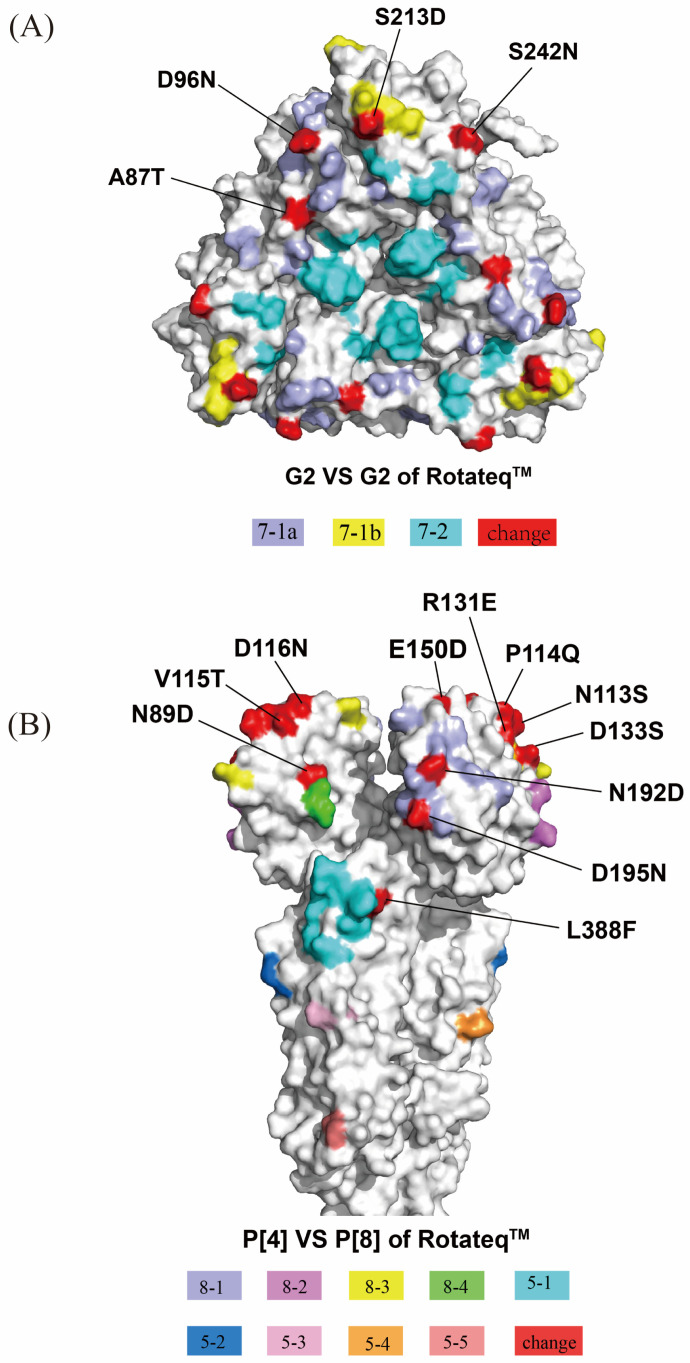
(**A**) Surface representation of the VP7 trimer (PDB 3FMG). Antigenic epitopes are shown in purple (7-1a), yellow (7-1b), and cyan (7-2). Surface-exposed residues that are different between circulating and RotaTeq strains are shown in red. (**B**) Surface representation of VP8* and VP5* (PDB 1KQR). Antigenic epitopes are shown in purple (8-1), violet (8-2), yellow (8-3), green (8-4), cyan (5-1), navy blue (5-2), light pink (5-3), orange (5-4), and pink (5-5). Surface-exposed residues that are different between circulating and RotaTeq strains are shown in red.

The critical epitopes of the VP7 protein are 7–1a, 7–1b and 7–2. The G2 lineage II strains had 92.6–92.8% identity with RotaTeq G2. Using the AA alignments for the VP7 proteins, we identified differences in these antigenic epitopes between the RVA strains and the cognate genes of the RotaTeq strains. The G2 strains showed four amino acid differences compared to RotaTeq G2. Among them, A87T, D96N (7–1a region), S213D, and S242N (7–1b region) may induce immunogenicity changes in the vaccine (Figure 4A and Table 3).

The VP4 spike protein undergoes proteolytic cleavage by trypsin-like proteases present in the gastrointestinal tract of the host into VP8* and VP5* subunits, which are the targets of neutralizing monoclonal antibodies [25]. The VP8* region contains four antigenic epitopes (8-1 to 8-4) composed of 25 AAs. The P[4] lineage V strains showed 86.2–87.1% identity with RotaTeq P[8]. All G2P[4] RVA strains showed differences in eight glycan-binding sites of the VP8* subunit (E150D, N192D, D195N, V115T, D116N, R131E, D133S, and N89D) (Figure 4). All strains except GS2260 showed amino acid differences at residue N113S in the 8–3 region. A neutralizing antigenic site mutation at residue 114 was found only in QD2210 and SX2205. The VP5* region has five epitopes (5-1 to 5-5) with 12 AAs. The sequence in the present study was highly conserved in the VP5* subunit compared to RotaTeq, with only GS2265 having an L388F amino acid difference in the 5-1 antigenic region (Figure 4B and Table 3).

## 4. Discussion

We conducted a genome-wide analysis of 13 G2P[4] genotype RVA strains from China in 2022 to identify epitope variations compared to vaccine antigens. Phylogenetic analysis revealed that the examined strains demonstrated characteristic features of typical G2P[4] DS-1-like strains in genetic clusters, with the exception of strain GS2265, which formed an independent cluster (E1) within the NSP4 segment.

From January to December 2022, 5360 samples were genotyped. G9P[8] was most predominant, followed by G8P[8], G2P[4], G1P[8], and G3P[8] (unpublished data from the National Viral Diarrhea Surveillance Network of China). The G2P[4] genotype is relatively rare in China. Phylogenetic comparison revealed that the study strains shared close genetic relationships with previously reported G2P[4] strains from mainland China [26,27], as well as from Japan [24], Russia [28], and Thailand [29]. Therefore, the G2P[4] strain in this study is prevalent globally. Previously, evidence was reported of interspecies transmission of the G2P[4] strain with the Chinese ruminant RVA strain in Vietnam [30]. However, the Chinese G2P[4] strain was not found to be associated with RVA in animals.

Phylogenetic analyses of 10 segments (excluding NSP4) revealed that QD2210 and SX2205 diverged from the other circulating RVA strains, clustering into two distinct sublineages. The structural (VP1-VP4, VP6, and VP7) and nonstructural (NSP1–NSP5) genes of QD2210 and SX2205 are most closely related at the nucleotide level to the Chinese Wuhan RVA/Human-wt/CHN/E6896/2021/G2P[4] strain. Epidemiological investigations revealed that cases GS2265 and GS2286 were geographically clustered within the same township. Notably, GS2265 developed RVA infection 4 months after the initial diagnosis of GS2286, accompanied by a genotype shift in NSP4 from E2 to E1. Phylogenetically, GS2265 showed close genetic relatedness to Wa-like strains circulating in China and neighboring countries. In addition, GS2265 represents a reassortant strain generated through a NSP4 gene segment reassort with a Wa-like strain, while retaining the DS-1-like genetic backbone.

There is currently no specific treatment for RVGE, and vaccination is the most effective measure to prevent RVGE [31]. The proteins encoded by the VP7 and VP4 genes contain a variety of antigenic epitopes that are key targets of the host immune response. Phylogenetic analysis of the VP7 gene showed that the RotaTeq G2 strains were in lineage III and the Chinese G2 strains were all in lineage II and were distantly related. Compared to RotaTeq, the Chinese G2 strain differed in four amino acid residues at the 7–1a and 7–1b antigenic epitopes, consistent with G2 strains detected in China (2016–2019) [32], Belgium [33], and Australia [34]. Alterations in A87T, D96N, and S213D, which can lead to altered vaccine immunogenicity [35], have been observed in most of the G2P[4] strains circulating worldwide, and changes in this combination are a stable feature of modern G2 strains. The protection of homotypic RVA vaccines is more effective than heterotypic varieties. Genetic variation in the epitope region of the VP7 antigen may affect the effectiveness of the RotaTeq vaccine against the G2P[4] strain [36]. Rotarix has 18 amino acid residues in the VP7 epitope that differ significantly from those of the G2 strains in this study. An analysis of phase II and III trial data for the monovalent rotavirus vaccine indicated that Rotarix is significantly less effective against fully heterotypic genotypes, specifically G2P[4], compared to its efficacy against G1P[8] (homotypic) and partially heterotypic genotypes [37]. Consequently, the monovalent vaccine was less effective in protecting against the G2 strains.

The strain in this study has a total of 10 amino acid residue mutations in the VP8* subunit compared to RotaTeq. Generally, VP4 is less susceptible to genetic mutation than VP7 because antibodies to VP8* are directly related to cellular receptor binding and can neutralize viral infection by inhibiting attachment and antibodies to VP5* and thus prevent membrane penetration [38]. However, the current study revealed L388F in region 5–1 in strain GS22655, which has not been reported previously in P[4] genotypes. The LLR vaccine strain produced in China is G10P[15] [39], showing significant amino acid differences from the G2P[4] strain. LLR3 was launched in China in 2023, and its sequence is not yet publicly available. Therefore, further amino acid level comparison between the Chinese G2P[4] strain and LLR3 was not performed. Nonetheless, LLR3 contains strains of G2, G3, and G4 [12], including the G2 genotype, which is hypothesized to enhance protection against the G2P[4] genotype.

In conclusion, the 13 RVA strains examined in this study belong to the G2P[4] genotype, which is prevalent worldwide. The G2P[4] genotype RVA in the Chinese population underwent reassortment with Wa-like strains for certain genes in the short term, forming new reassortant strains. The RotaTeq RV vaccine may have a superior preventive effect to Rotarix and LLR. LLR3 produces homotypic protection against G2 and may therefore more effectively protect against the G2P[4] genotype.

## Figures and Tables

**Table 1:** Case information of 13 G2P[4] RVA samples from China.

RVA Strain Name	Collection Region	Gender	Age in Months	Body Temperature (°C)	Diarrhea (Episodes/Day)	Vomiting (Episodes/Day)	Mixed Infection
RVA/Human-wt/CHN/GS2215/2022/G2P[4]	Gansu	male	13	37.3	4	3	None
RVA/Human-wt/CHN/GS2217/2022/G2P[4]	Gansu	male	7	36.5	7	3	None
RVA/Human-wt/CHN/GS2260/2022/G2P[4]	Gansu	female	6	38.5	3	/	Astrovirus
RVA/Human-wt/CHN/GS2265/2022/G2P[4]	Gansu	male	20	36.2	5	3	None
RVA/Human-wt/CHN/GS2285/2022/G2P[4]	Gansu	female	17	37.2	6	/	None
RVA/Human-wt/CHN/GS2286/2022/G2P[4]	Gansu	male	13	36.6	5	2	Norovirus
RVA/Human-wt/CHN/GS2287/2022/G2P[4]	Gansu	male	6	38.3	6	/	Norovirus
RVA/Human-wt/CHN/GD2253/2022/G2P[4]	Guangdong	male	16	36.9	4	/	None
RVA/Human-wt/CHN/JS2283/2022/G2P[4]	Jiangsu	female	25	36.1	4	4	None
RVA/Human-wt/CHN/HA2203/2022/G2P[4]	Henan	male	7	36.8	7	1	Norovirus, Astrovirus
RVA/Human-wt/CHN/HA2208/2022/G2P[4]	Henan	male	10	36.6	6	1	Norovirus, Astrovirus
RVA/Human-wt/CHN/QD2210/2022/G2P[4]	Shandong	male	11	37.9	3	2	None
RVA/Human-wt/CHN/SX2205/2022/G2P[4]	Shaanxi	male	/	/	/	/	/

Note: /, missing data. The location of the collection region is in the Supplementary Materials.

**Table 2:** Genotype constellation of the 11 segments of 13 G2P[4] RVA strains in China in 2022 and reference strains from the GenBank Database. To aid visualization, lineage constellations of representative G2P[4] genotype 2 strains are highlighted in various colors.

	VP7	VP4	VP6	VP1	VP2	VP3	NSP1	NSP2	NSP3	NSP4	NSP5
	G2	P[4]	I2	R2	C2	M2	A2	N2	T2	E2	H2
RVA/Human-wt/CHN/GS2215/2022/G2P[4]	IVa-3	IVa	V	V	IVa	VI	IVa	V	V	VI	IVa
RVA/Human-wt/CHN/GS2217/2022/G2P[4]	IVa-3	IVa	V	V	IVa	VI	IVa	V	V	VI	IVa
RVA/Human-wt/CHN/GS2260/2022/G2P[4]	IVa-3	IVa	V	V	IVa	VI	IVa	V	V	VI	IVa
RVA/Human-wt/CHN/GS2265/2022/G2P[4]	IVa-3	IVa	V	V	IVa	VI	IVa	V	V	E1	IVa
RVA/Human-wt/CHN/GS2285/2022/G2P[4]	IVa-3	IVa	V	V	IVa	VI	IVa	V	V	VI	IVa
RVA/Human-wt/CHN/GS2286/2022/G2P[4]	IVa-3	IVa	V	V	IVa	VI	IVa	V	V	VI	IVa
RVA/Human-wt/CHN/GS2287/2022/G2P[4]	IVa-3	IVa	V	V	IVa	VI	IVa	V	V	VI	IVa
RVA/Human-wt/CHN/GD2253/2022/G2P[4]	IVa-3	IVa	V	V	IVa	VI	IVa	V	V	VI	IVa
RVA/Human-wt/CHN/JS2283/2022/G2P[4]	IVa-3	IVa	V	V	IVa	VI	IVa	V	V	VI	IVa
RVA/Human-wt/CHN/HA2203/2022/G2P[4]	IVa-3	IVa	V	V	IVa	VI	IVa	V	V	VI	IVa
RVA/Human-wt/CHN/HA2208/2022/G2P[4]	IVa-3	IVa	V	V	IVa	VI	IVa	V	V	VI	IVa
RVA/Human-wt/CHN/QD2210/2022/G2P[4]	IVa-1	IVnon-a	V	V	IVa	V	IVa	V	V	VI	IVa
RVA/Human-wt/CHN/SX2205/2022/G2P[4]	IVa-1	IVnon-a	V	V	IVa	V	IVa	V	V	VI	IVa
RVA/Human-wt/CHN/Fuzhou21-23/2021/G2P[4]	IVa-1	IVnon-a	V	V	IVa	V	IVa	V	V	VI	IVa
RVA/Human-wt/CHN/E6896/2021/G2P[4]	IVa-1	IVnon-a	V	V	IVa	V	IVa	V	V	VI	IVa
RVA/Human-wt/JPN/Tokyo18-41/2018/G2P[4]	IVa-3	IVa	V	V	IVa	VI	IVa	V	V	VI	IVa
RVA/Human-wt/CHN/Pingtan21-2/2020/G2P[4]	IVa-3	IVa	V	V	IVa	VI	IVa	V	V	VI	IVa
RVA/Human-wt/RUS/NN67622/2022/G2P[8]	IVa-3	P[8]	V	V	IVa	VI	IVa	V	V	VI	IVa
RVA/Human-wt/ZWE/MRC-DPRU3347/2010/G8P[4]	G8	II	V	V	IVa	V	IVa	V	V	XXII	IVa
RVA/Human-wt/USA/VU10-119/2011/G2P[4]	IVa-3	IVa	V	V	IVa	V	IVa	V	V	VI	IVa
RVA/Human-tc/PHL/L26/1987/G12P[4]	G12	II	II	II	II	II	V	N1	II	II	H1
RVA/Human-wt/JPN/S120088/2012/G9P[4]	G9	IVa	V	V	IVa	V	IVa	V	T1	V	IVa
RVA/Human-tc/MWI/MW2-489/2000/G8P[4]	G8	II	V	II	IVa	V	IVa	V	V	V	IVa
RVA/Human-wt/JPN/KUN/1980/G2P[4]	III	III	III	III	III	III	III	III	III	III	III
RVA/Human-wt/JPN/89Y1520/1989/G2P[4]	IVa-1	IVnon-a	IV	IV	IVnon-a	IV	IVnon-a	N1	IV	IV	IVa
RVA/Human-wt/AUS/V233/1999/G2P[4]	IVa-3	IVa	V	V	IVa	V	IVa	V	V	V	IVa
RVA/Human-wt/VNM/NT0578/2008/G2P[4]	IVa-3	IVa	V	V	IVa	V	IVa	VIII	V	VIII	IVa
RVA/Human-wt/USA/06-242/2006/G2P[6]	IVa-3	P[6]	V	V	IVa	V	IVa	V	V	Distinct	IVa
RVA/Human/CHN/20200077/2020/G3P[8]	G3	P[8]	I1	R1	C1	M1	A1	N1	T1	E1	H1
RVA/Human-wt/CHN/TB-Chen/1996/G2P[4]	IVnon-a	III	IV	IV	IVnon-a	IV	IVnon-a	IV	Distinct	IV	IVnon-a
RVA/Human-wt/AUS/CK20001/1977/G2P[4]	I	I	I	I	I	II	I	I	I	I	I

**Table 3:** Alignment of the amino acid residues in VP7 and VP4 antigenic epitopes of the Chinese G2P[4] RVA strains and those of vaccine strains.

	Epitope 7-1a														Epitope 7-1b						Epitope 7-2								
	87	91	94	96	97	98	99	100	104	123	125	129	130	291	201	211	212	213	238	242	143	145	146	147	148	190	217	221	264
**G10 LLR**	**I**	**T**	**N**	**N**	**E**	**W**	**T**	**S**	**Q**	**N**	**A**	**V**	**D**	**K**	**Q**	**N**	**T**	**G**	**D**	**T**	**R**	**N**	**S**	**S**	**L**	**S**	**E**	**A**	**G**
**G1 Rotarix-A41CB052A**	**T**	**T**	**N**	**G**	**E**	**W**	**K**	**D**	**Q**	**S**	**V**	**V**	**D**	**K**	**Q**	**N**	**V**	**D**	**N**	**T**	**K**	**D**	**Q**	**N**	**L**	**S**	**M**	**N**	**G**
**G2 RotaTeq-SC2-9**	**A**	**N**	**S**	**D**	**E**	**W**	**E**	**N**	**Q**	**D**	**T**	**M**	**N**	**K**	**Q**	**D**	**V**	**S**	**N**	**S**	**R**	**D**	**N**	**T**	**S**	**D**	**I**	**S**	**G**
RVA/Human-wt/CHN/GS2215/2022/G2P[4]	T	N	S	N	E	W	E	N	Q	D	T	M	N	K	Q	D	V	D	N	N	R	D	N	T	S	D	I	S	G
RVA/Human-wt/CHN/GS2217/2022/G2P[4]	T	N	S	N	E	W	E	N	Q	D	T	M	N	K	Q	D	V	D	N	N	R	D	N	T	S	D	I	S	G
RVA/Human-wt/CHN/GS2260/2022/G2P[4]	T	N	S	N	E	W	E	N	Q	D	T	M	N	K	Q	D	V	D	N	N	R	D	N	T	S	D	I	S	G
RVA/Human-wt/CHN/GS2265/2022/G2P[4]	T	N	S	N	E	W	E	N	Q	D	T	M	N	K	Q	D	V	D	N	N	R	D	N	T	S	D	I	S	G
RVA/Human-wt/CHN/GS2285/2022/G2P[4]	T	N	S	N	E	W	E	N	Q	D	T	M	N	K	Q	D	V	D	N	N	R	D	N	T	S	D	I	S	G
RVA/Human-wt/CHN/GS2286/2022/G2P[4]	T	N	S	N	E	W	E	N	Q	D	T	M	N	K	Q	D	V	D	N	N	R	D	N	T	S	D	I	S	G
RVA/Human-wt/CHN/GS2287/2022/G2P[4]	T	N	S	N	E	W	E	N	Q	D	T	M	N	K	Q	D	V	D	N	N	R	D	N	T	S	D	I	S	G
RVA/Human-wt/CHN/GD2253/2022/G2P[4]	T	N	S	N	E	W	E	N	Q	D	T	M	N	K	Q	D	V	D	N	N	R	D	N	T	S	D	I	S	G
RVA/Human-wt/CHN/JS2283/2022/G2P[4]	T	N	S	N	E	W	E	N	Q	D	T	M	N	K	Q	D	V	D	N	N	R	D	N	T	S	D	I	S	G
RVA/Human-wt/CHN/HA2203/2022/G2P[4]	T	N	S	N	E	W	E	N	Q	D	T	M	N	K	Q	D	V	D	N	N	R	D	N	T	S	D	I	S	G
RVA/Human-wt/CHN/HA2208/2022/G2P[4]	T	N	S	N	E	W	E	N	Q	D	T	M	N	K	Q	D	V	D	N	N	R	D	N	T	S	D	I	S	G
RVA/Human-wt/CHN/QD2210/2022/G2P[4]	T	N	S	N	E	W	E	N	Q	D	T	M	N	K	Q	D	V	D	N	N	R	D	N	T	S	D	I	S	G
RVA/Human-wt/CHN/SX2205/2022/G2P[4]	T	N	S	N	E	W	E	N	Q	D	T	M	N	K	Q	D	V	D	N	N	R	D	N	T	S	D	I	S	G
					▲	▲			▲					▲	▲														▲
	**8-1**															**8-2**	**8-3**										**8-4**		
	**100**			**146**			**148**	**150**	**188**	**190**	**192**	**193**	**194**	**195**	**196**	**180**	**183**	**113**	**114**	**115**	**116**	**125**	**131**	**132**	**133**	**135**	**87**	**88**	**89**
**P[15] LLR**	**D**			**T**			**A**	**G**	**Y**	**S**	**T**	**N**	**Y**	**D**	**S**	**E**	**N**	**P**	**E**	**T**	**T**	**T**	**A**	**N**	**P**	**Q**	**T**	**S**	**E**
**P[8] Rotarix-A41CB052A**	**D**			**S**			**Q**	**E**	**S**	**T**	**N**	**L**	**N**	**N**	**I**	**T**	**A**	**N**	**P**	**V**	**D**	**S**	**S**	**N**	**D**	**N**	**N**	**T**	**N**
**P[8] RotaTeq-WI79-4**	**D**			**S**			**Q**	**E**	**S**	**T**	**N**	**L**	**N**	**D**	**I**	**T**	**A**	**N**	**P**	**V**	**D**	**N**	**R**	**N**	**D**	**D**	**N**	**T**	**N**
RVA/Human-wt/CHN/GS2215/2022/G2P[4]	D			S			Q	D	S	T	D	L	N	N	I	T	A	S	P	T	N	N	E	N	S	D	N	T	D
RVA/Human-wt/CHN/GS2217/2022/G2P[4]	D			S			Q	D	S	T	D	L	N	N	I	T	A	S	P	T	N	N	E	N	S	D	N	T	D
RVA/Human-wt/CHN/GS2260/2022/G2P[4]	D			S			Q	D	S	T	D	L	N	N	I	T	A	N	P	T	N	N	E	N	S	D	N	T	D
RVA/Human-wt/CHN/GS2265/2022/G2P[4]	D			S			Q	D	S	T	D	L	N	N	I	T	A	S	P	T	N	N	E	N	S	D	N	T	D
RVA/Human-wt/CHN/GS2285/2022/G2P[4]	D			S			Q	D	S	T	D	L	N	N	I	T	A	S	P	T	N	N	E	N	S	D	N	T	D
RVA/Human-wt/CHN/GS2286/2022/G2P[4]	D			S			Q	D	S	T	D	L	N	N	I	T	A	S	P	T	N	N	E	N	S	D	N	T	D
RVA/Human-wt/CHN/GS2287/2022/G2P[4]	D			S			Q	D	S	T	D	L	N	N	I	T	A	S	P	T	N	N	E	N	S	D	N	T	D
RVA/Human-wt/CHN/GD2253/2022/G2P[4]	D			S			Q	D	S	T	D	L	N	N	I	T	A	S	P	T	N	N	E	N	S	D	N	T	D
RVA/Human-wt/CHN/JS2283/2022/G2P[4]	D			S			Q	D	S	T	D	L	N	N	I	T	A	S	P	T	N	N	E	N	G	D	N	T	D
RVA/Human-wt/CHN/HA2203/2022/G2P[4]	D			S			Q	D	S	T	D	L	N	N	I	T	A	S	P	T	N	N	E	N	S	D	N	T	D
RVA/Human-wt/CHN/HA2208/2022/G2P[4]	D			S			Q	D	S	T	D	L	N	N	I	T	A	S	P	T	N	N	E	N	S	D	N	T	D
RVA/Human-wt/CHN/QD2210/2022/G2P[4]	D			S			Q	D	S	T	D	L	N	N	I	T	A	S	Q	T	N	N	E	N	S	D	N	T	D
RVA/Human-wt/CHN/SX2205/2022/G2P[4]	D			S			Q	D	S	T	D	L	N	N	I	T	A	S	Q	T	N	N	E	N	S	D	N	T	D
	▲																												
	**5-1**																									**5-2**	**5-3**	**5-4**	**5-5**
	**384**						**386**			**388**				**393**		**394**		**398**		**440**		**441**				**434**	**459**	**429**	**306**
**P[15] LLR**	**T**						**N**			**Q**				**Q**		**W**		**S**		**S**		**R**				**E**	**R**	**S**	**I**
**P[8] Rotarix-A41CB052A**	**Y**						**F**			**I**				**W**		**P**		**G**		**R**		**T**				**P**	**E**	**L**	**R**
**P[8] RotaTeq-WI79-4**	**Y**						**F**			**L**				**W**		**P**		**G**		**R**		**T**				**P**	**E**	**L**	**R**
RVA/Human-wt/CHN/GS2215/2022/G2P[4]	Y						F			L				W		P		G		R		T				P	E	L	R
RVA/Human-wt/CHN/GS2217/2022/G2P[4]	Y						F			L				W		P		G		R		T				P	E	L	R
RVA/Human-wt/CHN/GS2260/2022/G2P[4]	Y						F			L				W		P		G		R		T				P	E	L	R
RVA/Human-wt/CHN/GS2265/2022/G2P[4]	Y						F			F				W		P		G		R		T				P	E	L	R
RVA/Human-wt/CHN/GS2285/2022/G2P[4]	Y						F			L				W		P		G		R		T				P	E	L	R
RVA/Human-wt/CHN/GS2286/2022/G2P[4]	Y						F			L				W		P		G		R		T				P	E	L	R
RVA/Human-wt/CHN/GS2287/2022/G2P[4]	Y						F			L				W		P		G		R		T				P	E	L	R
RVA/Human-wt/CHN/GD2253/2022/G2P[4]	Y						F			L				W		P		G		R		T				P	E	L	R
RVA/Human-wt/CHN/JS2283/2022/G2P[4]	Y						F			L				W		P		G		R		T				P	E	L	R
RVA/Human-wt/CHN/HA2203/2022/G2P[4]	Y						F			L				W		P		G		R		T				P	E	L	R
RVA/Human-wt/CHN/HA2208/2022/G2P[4]	Y						F			L				W		P		G		R		T				P	E	L	R
RVA/Human-wt/CHN/QD2210/2022/G2P[4]	Y						F			L				W		P		G		R		T				P	E	L	R
RVA/Human-wt/CHN/SX2205/2022/G2P[4]	Y						F			L				W		P		G		R		T				P	E	L	R

Note: Sites different from RotaTeq are indicated in blue. ▲, residues identical to all three vaccines.

## Data Availability

The datasets used and analyzed during the current study are available from the corresponding author on reasonable request.

## Supplementary Materials

The following supporting information can be downloaded at: https://www.mdpi.com/article/10.3390/v17030326/s1, Figure S1: The location of the collection region for the samples of this study.
