# Supplementary figures and images for: Whole-Genome Analysis of G2P[4] Rotavirus Strains in China in 2022 and Comparison of Their Antigenic Epitopes with Vaccine Strains
Source: Viruses. 2025 Feb 26;17(3):326. doi: 10.3390/v17030326 (PMC11945518; doi:10.3390/v17030326)

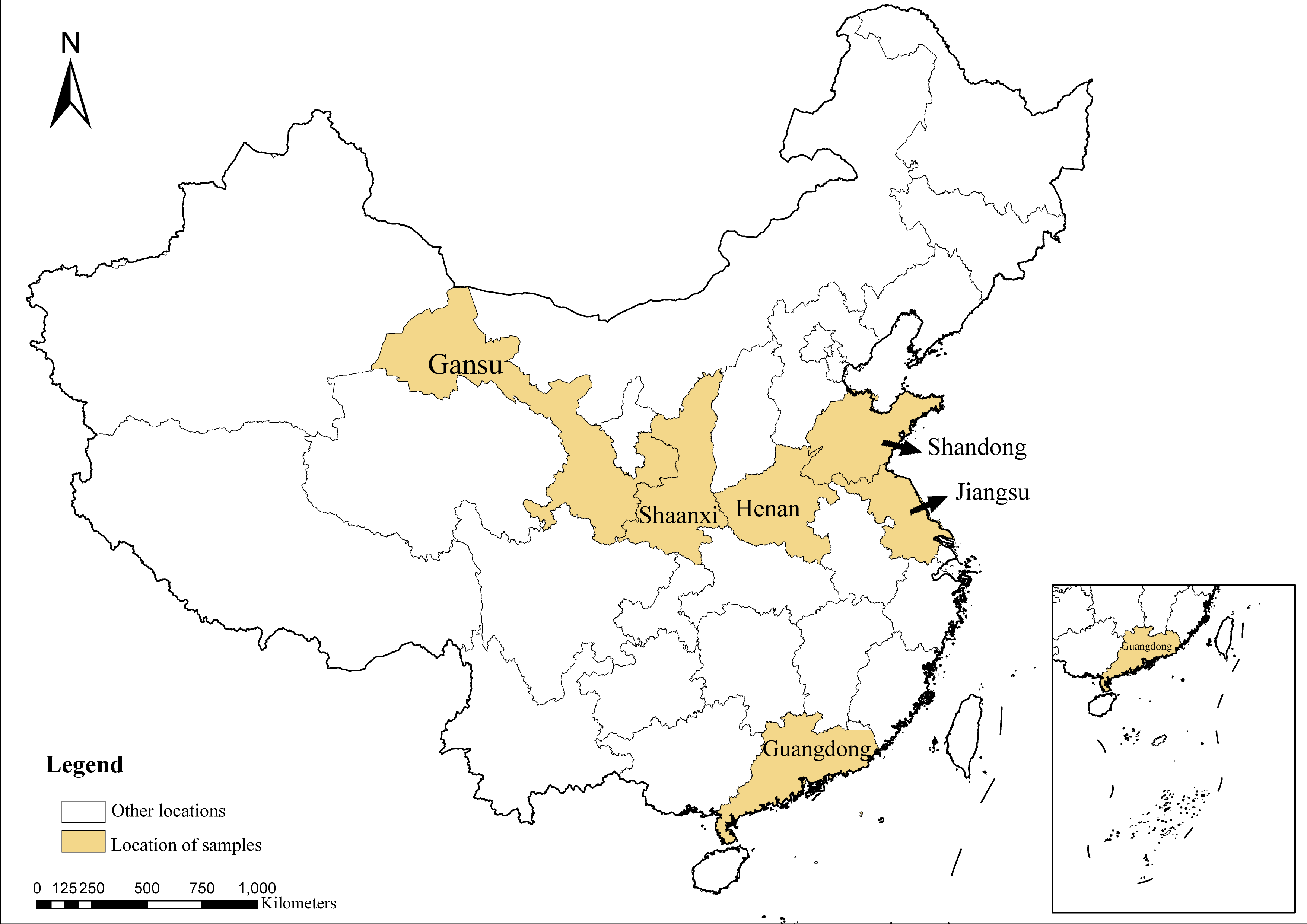

Supplement: Supplementary file 1 [file viruses-17-00326-s001.zip › viruses-3421607-supplementary.tif]
